# Endoscopic, Single-Session Management of Encrusted, Forgotten Ureteral Stents

**DOI:** 10.3390/medicina55030058

**Published:** 2019-02-26

**Authors:** Volkan Ulker, Orcun Celik

**Affiliations:** Department of Urology, Tepecik Training and Research Hospital, Health Sciences University, 35180 Izmir, Turkey; orcuncelik82@hotmail.com

**Keywords:** forgotten ureteral stent, extracorporeal shockwave lithotripsy, percutaneous nephrolithotomy, ureteroscopy

## Abstract

*Background and Objectives:* Remained or forgotten ureteral double-J stents may cause serious complications. Removing of an encrusted, forgotten stent can be challenging. We present our experience with heavily encrusted ureteral stents and discuss the endourologic treatment options as well as their effectivity. *Materials and Methods:* Eleven men and six women (mean 48.58 ± 14.48 years of age) with 18 encrusted forgotten stents (mean 16.4 ± 13.25 months of indwelling) were treated at our clinic. All patients underwent the operation after negative urine cultures were obtained. Their medical records were retrospectively reviewed and analyzed in terms of number of interventions required to remove the stent, operation time, complications, hospital stay and stone-free rate. *Results:* According to the Forgotten-Encrusted-Calcified (FECal) classification, the most common form of stent encrustation was grade III (64.7%) and 17.6% of the stents were fragmented. Four of 17 patients were initially treated with extracorporeal shock-wave lithotripsy. The patients required a mean of two endoscopic interventions for removing the encrusted stent and all stents were removed endoscopically in a single session. The mean operating time was 63.3 ± 41.8 minutes. Cystolithotripsy followed by ureteroscopy was the most common intervention (41.1%). Of the 17 patients, peroperative and postoperative complications were Clavien grade I in two, grade II in two and grade IIIb in one. The mean hospital stay was 1.3 ± 0.99 days. All patients were stone-free after a month of stent removal. *Conclusions:* The endourological removal of the encrusted forgotten stents in a single session is feasible and effective with a minimal morbidity. The treatment strategy should be to minimize the number of interventions.

## 1. Introduction

Ureteral double-J stents (DJSs) are extensively used in modern urological practice. DJS placement is indicated in treatment of urinary stone disease, to relieve benign or malign obstruction, and to promote ureteral healing and manage urinary leaks [1]. The main purpose of their use is to maintain drainage of the upper urinary system. Today, the most common use of DJS is after ureterorenoscopic treatment of urinary stones. DJSs are mostly placed for temporary purposes and need to be removed on their maximal safe life depending on their production material or coating. Despite their common use, 12% of DJSs are retained or forgotten for different reasons. Forgotten ureteral stents (FUS) may lead to infection, migration, encrustation and fragmentation [2,3]. Furthermore, more serious complications such as sepsis, renal failure [4] or even mortality have been reported with encrusted and infected FUS [5].

Severe encrustation may not allow a DJS to be removed cystoscopically. Removal of a heavily encrusted FUS may require combined endourologic procedures or open surgery and may represent a challenge for urologists [6,7]. However, there are no guidelines for the optimal management algorithm of encrusted FUS. Extra-corporeal shock wave lithotripsy (ESWL), ureteroscopy (URS), and percutaneous nephrolithotomy (PCNL) have been reported as treatment methods of encrusted stents [8,9,10,11]. In this study, we present our patients who had encrusted DJSs and discuss the endourological treatment options, their effectivity and safety.

## 2. Materials and Methods

Between January 2013 and June 2018, a total of 17 patients with 18 impacted and encrusted DJS were treated in our clinic. After approval of institutional ethics committee (2018/3-5, 04.08.2018), a retrospective review and analysis was performed for the patients who were treated in this period. The definition for inclusion was a DJS that could not be removed cystoscopically. The primary outcome was removal of the encrusted DJS. Secondary outcomes were number of interventions for removing the stent, operation time, peroperative and postoperative complications, hospital stay and stone-free rate. All patients were preoperatively evaluated by kidney-urinary-bladder (KUB) graphy, computed tomography (CT) with contrast and urine culture. In patients with poor or non-visualized kidneys on CT-urography, Tc99^m^ diethylene triamine penta acetic-acid (DTPA) renal sintigraphy was used to estimate the renal function. The degree of encrustation was cathegorized according to the FECal (forgotten-encrusted-calcified) classification, which was described by Acosta-Miranda et al. [12]. In this classification; grade I: minimal linear encrustations along either of the pigtail portions of the indwelling ureteral stent, grade II: minimal linear encrustations along either of the pigtail portions of the indwelling ureteral stent, grade III: circular encrustation completely encasing either of the pigtail portions as well as linear encrustation of the ureteral aspects of the indwelling ureteral stent, grade IV: circular encrustations completely encasing both of the pigtail portions of the indwelling ureteral stent, and grade V: diffuse and bulky encrustations completely encasing both of the pigtail and ureteral portions of the indwelling ureteral stent, were described, respectively. 

**Figure 1:**
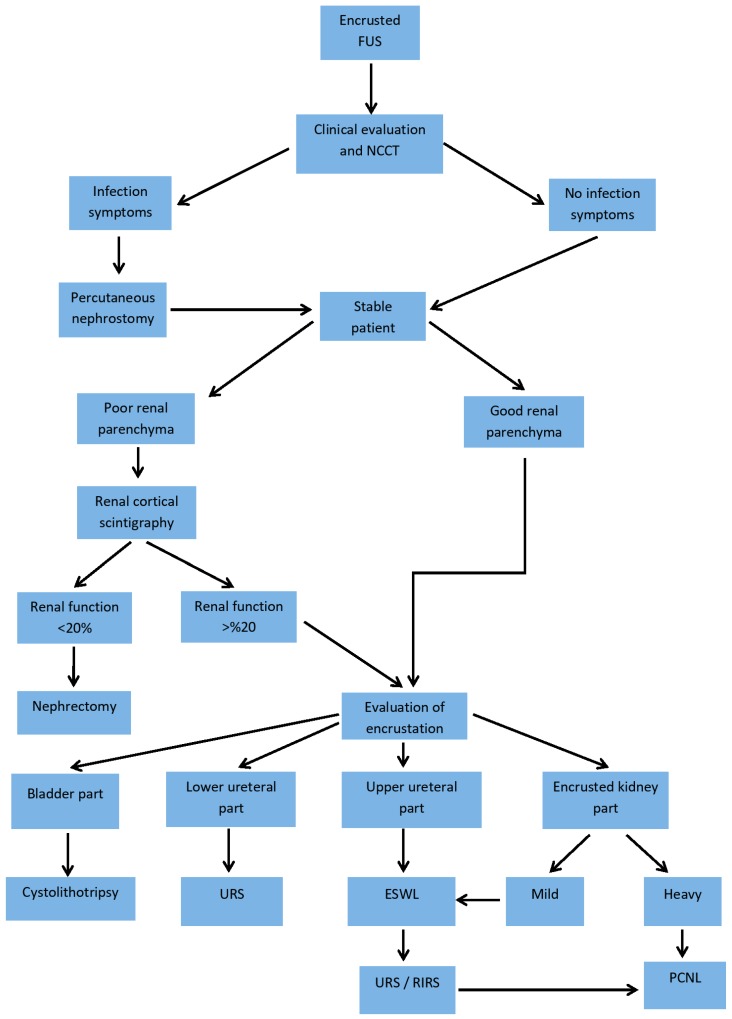
Treatment algorithm.

Treatment decisions were based on radiological findings and clinical presentations (Figure 1). Patients with positive urine cultures were treated preoperatively with appropriate antibiotics according to antibiogram results, and negative urine cultures were obtained in all patients before surgery. The ESWL treatment was performed under intravenous sedation using EMD E-1000 electrohydrolic generator (EMD Medical, Ankara, Turkey) and 1200–1500 shocks were applied ranging from 15.5 to 17.5 kV. The DJSs of patients, which were determined to be free of encrustation on KUB after ESWL, were removed under local anesthesia with a flexible cystoscope. All endoscopic surgical procedures were performed under general anesthesia in one session. Antibiotic prophylaxis with first generation cephalosporins was administered preoperatively to all patients. Cystoscopy was performed with a 22 French (F) cystoscope (Karl Storz, Germany). For the bladder parts of encrusted stents, cystolithotripsy was performed with 30 watt holmium:yttrium-aluminum-garnet (Ho:YAG) laser (Litho-laser, IML, Portland, OR, USA) and 550 micron (µ) laser probe. In the case of encrusted ureteral part, 7F semi-rigid ureteroscope (Karl Storz Tuttlingen, Germany) was inserted to the ureter following 0.35 inch guide-wire (Sensor, Boston Scientific, Marlborough, MA, USA) placement, and then lithotripsy was performed with 365µ laser probe until reaching to the proximal ureter and releasing the DJS. If the proximal part of DJS was encrusted, lithotripsy was performed with flexible URS through a guide-wire (Flex-X^2^, Karl Storz, Germany) and a 272µ laser probe, and then the DJS was removed with a grasping forceps. The Ho:YAG laser settings were 0.5–1.2 Joule and 8–50 Hertz. In the case of heavily encrusted proximal part or large stone formation, PCNL was performed in a prone position with nephroscope (Karl Storz, Germany) and encrusted DJS was removed after ultrasonic or pneumatic lithotripsy (Swiss Lithoclast Master, EMS, Nyon, Switzerland). Peroperative and postoperative complications were graded according to the Clavien–Dindo classification [13]. Postoperatively, stone-free and stent-free status was confirmed initially by KUB and then with non-contrast CT after four weeks of surgery as our routine. Stone-free status was defined for no residuel stone fragments larger than 4 mm on CT. The reason for DJS placement and indwelling time, surgical technique, total operation time, peroperative and postoperative complications and stone-free status of the patients were retrospectively evaluated. 

## 3. Results

**Figure 2:**
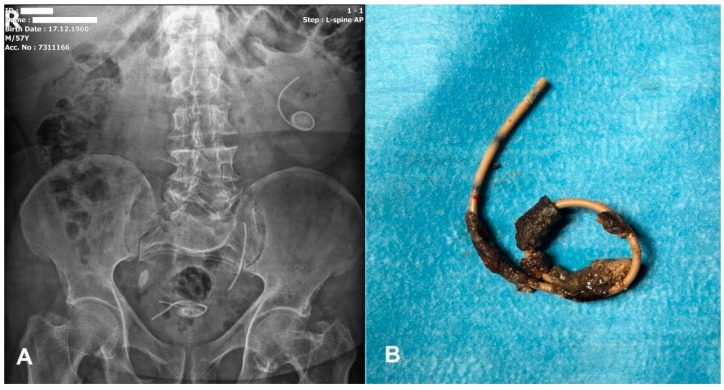
Encrusted and triple fragmented FUS (forgotten ureteral stents), which was retained for 34 months. (**A**) KUB (kidney-urinary-bladder) graphy; (**B**) proximal part of the encrusted DJS (double-J stents) which was removed by PCNL (percutaneous nephrolithotomy).

Eleven men and six women with a mean age of 48.58 ± 14.48 years (mean ± SD, range 23 to 72 years) were treated for encrusted FUS in our clinic. The patients’ demographics and treatment characteristics are shown in Table 1. The mean indwelling time of stents was 16.4 ± 13.25 months (ranging from 8 to 60 months) and the most common form of encrustation was FECal grade III (66.6%). Of the stents, 44.5% was on the right and 55.5% was on the left. All removed stents were polyurethane with a maximal safe life of three months. Twelve of 17 (70.5%) stents had been placed in different centers other than our hospital. Two patients (11.7%) had grade II hydronephrosis on the stented side, probably due to occlusion of the stent lumen. However, no one of them had renal deterioration or non-functioning kidneys on DMSA. In three patients (17.6%), stents were fragmented into two or three pieces (Figure 2) and, in one patient, (5.8%) the stent had migrated to upward and encrusted. One patient had a solitary kidney. Preoperative urine culture was positive in 29.4% of the patients. 

Five of 17 patients (29.4%) who had encrustation on the ureteral or kidney parts of the stent were initially treated with two to three sessions of ESWL before endoscopic removal. The patients required one to three endoscopic surgical interventions (mean 1.8 endourologic interventions per patient) and all interventions were completed in one session (Table 2). Cystolithotripsy followed by URS was the most common used treatment modality. The removed DJS was replaced with a new one in six of 17 patients (35.2%). In two patients, PCNL was required to remove the heavily encrusted upper part of the stent. In these patients, a nephrostomy tube was removed in the postoperative second day. Intraoperatively, ureteral mucosal injury (Clavien grade I) was observed in two patients during URS and was simply managed by the placement of DJS. Postoperative fever, which lasted 24 hours, was observed in two patients (Clavien grade II). Postoperatively, hematuria (Clavien grade I) in three patients and de novo stent migration (Clavien IIIb) in one patient after URS were observed. This upward migrated stent was removed with URS and replaced with a new DJS in the postoperative second day. The mean operating time was 63.3 ± 41.8 minutes. All the encrusted stents were removed successfully in a single session. In 7 of 17 (41.1%) patients, multiple residual stone fragments smaller than 5 mm were observed in KUB. However, after four weeks, these patients became stone-free with medical treatment including alpha-blockers and non-steroidal anti-inflammatory drugs. The mean hospital stay was 1.3 ± 0.99 days (range 1–4 days). Stone analysis could be done in two patients (11.7%) only. Analysis resulted as calcium phosphate in one and calcium oxalate in the other patient.

## 4. Discussion

Despite their extensive use, DJSs may cause severe discomfort and morbidity including lumbar pain, hematuria, dysuria and lower urinary tract symptoms [14]. On the other hand, technological advances in DJS design and materials allow patients to tolerate DJS more easily and this may cause a decrease in patient compliance for the removal. Patients may forget their in situ stents because they have no stent related complaints or neglect to apply to a hospital for stent removal since his or her doctor did not provide enough information about the DJS. The reasons of FUS are not clear. Divakaruni et al. reported that females and patients without medical insurance had a higher risk of FUS [2]. It has been shown that there is a correlation between stent indwelling time and encrustation. El-Faqih et al. reported that encrustation occurred in 9.2% of the stents under six weeks, 47.5% between 6–12 weeks and 76.3% after 12 weeks in removed DJSs which were placed for urinary stone disease [15]. Other reported risk factors for stent encrustation include history of urinary tract infection [16], urolithiasis [17] and pregnancy [18].

Internal ureteral stents offer an ideal surface for bacterial colonization and biofilm formation [19,20]. This biofilm formation leads to encrustation on the outer and inner surface of the DJS. Urinary pH also plays an important role. Enzyme urease, which was produced by bacteria such as *Proteus mirabilis* and *Pseudomonas* species, splits urea into ammonia and increased urinary pH causes precipitation of magnesium ammonium phosphate (struvite) and calcium phosphate crystals. These crystals lead to incrustation and mineralization of biofilm layer on the stent. In this biofilm environment, microorganisms appear to be more resistant to antimicrobial agents [21]. Encrustation can occur in sterile or infected urine due to a combination of urinary pH, bacterial enzymes, and biomaterial. Another important complication of encrusted FUS is fragmentation. Fragmentation rate in FUS has been reported between 0.3–10% [7,15]. In our cases, stent fragmentation rate was 11.7%. Stent encrustation and fragmentation are probably related to the biomaterial of DJS. It has been shown that silicon had the least encrustation potential compared to polyurethan and hydrophilic coated polyurethan materials [22].

Previously, some classifications of stent encrustation have been presented. Singh et al. described a classification based on encrustation volume. In this classification, stent encrustation was graded as mild (<100 mm^2^), moderate (100–400 mm^2^) and severe (>400 mm^2^). However, this classification did not take into account the location of encrustation [23]. Similarly, Arenas et al. reported a scoring system in which encrustation of the stent was graded separately as in the bladder, ureter, and kidney parts [24]. They concluded that, with this scoring system, encrusted stents requiring multiple surgeries and multimodal surgery could be identified and operation time could be estimated. In our study, we applied the classification that was described by Acosta-Miranda et al. [12]. Although this classification does not include some scenarios, such as encrustation only on the ureteral portion of the stent, it is simple and easy to use.

Management of an encrusted DJS can be quite difficult. There are no clear treatment guidelines or consensus for the surgical removal of encrusted DJS. However, some surgical treatment algorithms have been formed according to the place and rate of encrustation [10,25]. Based on FECal classification, grade I encrusted stents can be removed with cystoscopy or with cystolithotripsy of the distal part only. Grade II, proximally encrusted stents can be removed after ESWL on this part. On the other hand, grades III, IV and V encrusted stents usually require combined endourological interventions, including URS, RIRS (retrograd intrarenal surgery) and PCNL. Rarely, open or laparoscopic surgery is needed for removal of the heavily encrusted DJSs [6,7,26]. Proximal stone burden of the stent has been described as a main determining factor in the management of encrusted FUS and correlated with multiple surgeries and surgical complications [27]. It should be kept in mind that KUB may underestimate the proximal stone burden, especially in radiolucent stones. Therefore, surgical planning should be done with CT. ESWL could be recommended initially for mild to moderate encrustations on the proximal ureteral or kidney parts of the FUS. However, ESWL is indicated when renal function is normal to achieve clearance of stone fragments [24]. After initial ESWL, if it is needed, we prefer to complete releasing of the lower segment first. Previously reported studies also support this approach [11,18].

**Figure 3:**
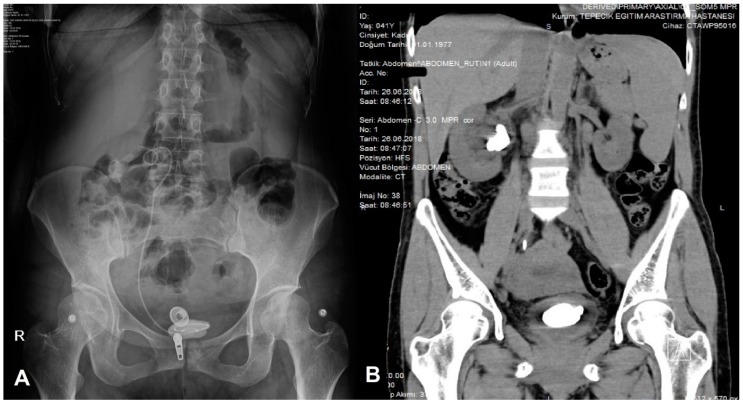
Heavily proximal and distal encrustation and stone formation of an FUS, which retained for 28 months. (**A**) KUB film; (**B**) non-contrast CT (computed tomography).

We succeded in removing 76.6% of encrusted FUS with a combination of cystolithotripsy and URS or URS alone without any major complication. During URS, no force should be used to remove encrusted stent since ureteral injury or stent fragmentation can easily occur. In cases of moderate encrustation of proximal segments, flexible URS and RIRS in combination with Ho:YAG laser represents a minimally-invasive treatment alternative if ESWL fails (Figure 3). However, access to the upper urinary system usually can be done through a guide-wire since a ureteral access sheath (UAS) may not accommodate the ureter together with DJS. Thomas et al. suggested cutting the stent using Ho:YAG to allow insertion of the UAS [28]. Aravantinos considered that placing a second DJS for passive dilation and completing the RIRS through an UAS in a second-session could be a reasonable approach [10]. Our treatment of choice was PCNL for large stone burden on the upper segment. Additionally, PCNL can be the only option to remove a fragmented proximal segment like in our case (Figure 3). Pais et al. reported in their 36-patient series that PCNL alone was sufficient to remove encrusted FUS only in 21% of cases [9]. When combined with other endourologic procedures, they succeeded in removing all encrusted DJSs. Bultitude and colleagues used PCNL as a second-line treatment modality [6]. We needed to perform PCNL only in two patients and their heavily encrusted stents were removed successfully in the same session. No blood transfusions were required and no major complications were noted.

Overall, we could successfully remove all encrusted stents in a single endourologic session in a reasonable operating time with minimal morbidity. Peroperative and postoperative complications were mostly Clavien grades I–II and were easily managed conservativelly. No open or laparoscopic surgery was needed to remove an encrusted stent in our series. After four weeks of postoperative period, all patients were stone-free on CT. Our results support the previously reported studies which showed that single session endourologic surgery was feasible for the management of encrusted FUS [9,10,25,28].

The present study has some limitations, such as the retrospective nature of the file review and relatively small number of patients. Similarly, our study contains a proportionally small number of severe, FECal grade V cases, which might lead to getting better results. In addition, we were able to perform stone analysis only in a few patients. Finally, the lack of long-term follow-up results is among the limitations of this study.

## 5. Conclusions

Our study results showed that the endourological treatment of encrusted forgotten ureteral stents in a single session was feasible and safe. The treatment strategy should be to minimize the number of interventions. Technological advances and miniaturization of endoscopic instruments facilitated the endoscopic treatment of encrusted FUS. Although we can effectively treat patients with encrusted FUS endourologicaly, the best treatment remaining is prevention. 

## Figures and Tables

**Table 1:** Patients’ demographics and treatment characteristics.

N	Age/Sex	Side	Reason for Stenting	Indwelling Time (months)	FECal Grade	Fragmentation	Treatment Modality	Operation Time (min)	Hospital Stay (days)
1	50/M	L	Retroperitoneal fibrosis	15	III	−	CL + URS	65	1
2	48/M	R	After URS	8	IV	−	ESWL + CL	25	1
3	63/F	L	After URS	12	III	−	ESWL + URS	35	1
4	55/M	R	Renal transplantation	60	III		CL + URS	50	2
5	41/F	L	Ureteral stone	8	III	−	URS	40	1
6	34/F	L	After URS	15	V	+	CL + URS + RIRS	130	1
7	32/M	L	After URS	11	III	−	CL + URS	25	1
8	23/F	R	Hydronephrosis in pregnancy	10	IV	−	CL + RIRS	100	2
9	54/M	R	After URS	34	V	−	CL + URS + PCNL	155	3
10	61/M	L	After URS	16	III	−	ESWL + URS	35	1
11	59/M	L	Ureteral stone	9	III	−	CL + URS	50	2
12	72/F	R	After URS	28	V	+	CL + URS + PCNL	120	4
13	68/M	R, L	After URS	12	III, III	−	CL + URS	40	1
14	30/F	L	Hydronephrosis in pregnancy	9	IV	−	ESWL + CL	20	1
15	33/M	R	After URS	10	III	−	CL + URS	35	1
16	58/M	L	Retroperitonel fibrosis	12	III	−	ESWL + URS	25	1
17	45/F	R (solitary)	After URS	10	III	−	CL + URS	45	1

CL = cystolithotriopsy, URS = ureteroscopy, ESWL = extra-corporeal shock wave lithotripsy, RIRS = retrograd intrarenal surgery, PCNL = percutaneous nephrolithotomy, M = male, F = female L = left, R = right.

**Table 2:** Endoscopic treatments used for removing encrusted FUS.

Treatment Modality	*n* (%)
URS after ESWL	3 (16.7)
Cystolithotripsy after ESWL	2 (11.1)
URS alone	1 (5.5)
Cystolithotripsy + URS	7 (44.5)
Cystolithotripsy + URS + RIRS	1 (5.5)
Cystolithotripsy + RIRS	1 (5.5)
Cystolithotripsy + URS + PCNL	2 (11.1)
